# The association in a two-way contingency table through log odds ratio analysis: the case of Sarno river pollution

**DOI:** 10.1186/2193-1801-3-384

**Published:** 2014-07-28

**Authors:** Ida Camminatiello, Antonello D’Ambra, Pasquale Sarnacchiaro

**Affiliations:** Department of Economics, Second University of Naples, Corso Gran Priorato di Malta, 81043 Capua, CE, Italy; University of Rome Unitelma Sapienza, Viale Regina Elena 295, 00161 Rome, Italy

**Keywords:** Log ratio analysis, Weighted log odds ratio analysis, Association measures, Two-way contingency table, Water pollution

## Abstract

In this paper we are proposing a general framework for the analysis of the complete set of log Odds Ratios (ORs) generated by a two-way contingency table. Starting from the RC (M) association model and hypothesizing a Poisson distribution for the counts of the two-way contingency table we are obtaining the weighted Log Ratio Analysis that we are extending to the study of log ORs. Particularly we are obtaining an indirect representation of the log ORs and some synthesis measures. Then for studying the matrix of log ORs we are performing a generalized Singular Value Decomposition that allows us to obtain a direct representation of log ORs. We also expect to get summary measures of association too. We have considered the matrix of complete set of ORs, because, it is linked to the two-way contingency table in terms of variance and it allows us to represent all the ORs on a factorial plan. Finally, a two-way contingency table, which crosses pollution of the Sarno river and sampling points, is to be analyzed to illustrate the proposed framework.

## Introduction

Polycyclic aromatic hydrocarbons (PAHs) are a group of lipophilic contaminants widespread in the environment. This class of compounds has been widely studied (Tolosa et al., 1995; Caricchia et al., 1993) because of its carcinogenic and mutagenic properties (Lehr and Jerima, 1997; Yan, 1985; White 1986).

PAHs are produced by both anthropogenic and natural processes and can be introduced into the environment through various routes. Anthropogenic inputs can originate from the incomplete combustion of organic matter (pyrolytic) and the discharge of crude oil-related material (petrogenic). PAHs can also originate from natural processes such as short-term diagenetic degradation of biogenic precursors (diagenesis). Each source (i.e. pyrolytic, petrogenic and diagenetic) gives rise to characteristic PAH patterns. Currently, the interest in multivariate statistical methodologies for identifying the main sources of PAH pollution and for quantifying the incidence of each source of pollution on total pollution levels, particularly in coastal environments, is increasing (Luo et al., 2006; Bihari et al., 2007; Xu et al., 2007; Sarnacchiaro et al., 2012). This study is part of a large project which has the objective of enhancing the knowledge of pollution in the Sarno River and its environmental impact on the gulf of Naples. This project has attempted to assess the pollution derived from local industries, agriculture and urban impact (Sarnacchiaro et al., 2012). In the present work, we have studied the association between the level of PAH pollution and the sampling points.

The analysis of the association for variables placed in a *I × J* two-way contingency table is a topic widely discussed. In this paper we focus our attention on the Odds Ratios (ORs) as measure of association. In a two-way contingency table the total number of ORs, that can be computed, may be too large, for their synthesis four main alternatives or complementary strategies have been performed. The first consists in the computation of statistical measures (Altham, 1970). The second is based on the construction of the model for frequencies and studies the ORs through the interaction between the row and column variables. The log-linear model for two-way contingency table belongs to this class. The third solution is the RC (M) association model (Goodman, 1985), which is more parsimonious than the usual log-linear model (Choulakian, 1988). The fourth strategy takes in consideration Singular Value Decomposition (SVD) of the matrix containing the basic set of log ORs (de Rooij and Anderson 2007).

In this paper we have proposed a general framework for the analysis of the complete set of log ORs generated by a two-way contingency table. The road map of the general framework is the following: we started from the RC (M) association model and we hypothesized a Poisson distribution for the counts of the two-way contingency table. Then for parameter estimations of RC (M) we use an alternative approach based on least squares. The matrix for the estimation of bilinear part of RC (M) has been linked to the matrix used in log-ratio analysis (Greenacre, 2009), moreover we have extended log-ratio analysis (LRA) to the study of log ORs. Then we have connected these methodologies with De Rooiij and Anderson’s approach and we have introduced some important properties that have allowed us to have deeper knowledge on the association between variables troughs ORs. Differently from De Rooiij and Anderson, in our approach we have chosen to consider the matrix of a complete set of ORs, because, as we will show, this matrix is linked to the two-way contingency table and it allows us to represent all the ORs on a factorial plan. Moreover, the spanning cell odds ratios are useful when one of the categories defines a control or reference group. In that case, all other categories are described against this reference group. The local odds ratios are useful for ordinal variables when all local odds ratios are larger or equal to 1 (de Rooij and Anderson 2007).

## Materials and methods

### The research plan - study area, sampling points

Nicknamed “the most polluted river in Europe”, the Sarno River originates in south-western Italy and has a watershed of about 715 km^2^. An intensive sampling campaign was conducted in the spring of 2008. Surface sediment samples were collected at four locations along the Sarno river (near the source of the river, just before and after the junction with Alveo Comune and at the river mouth) and nine points in the continental shelf around the river mouth (three points, one for each direction North-West, West and South-West, were sampled 50 m from the Sarno River mouth, another three points 150 m away and, finally, another three points 500 m from the river mouth). The collected data were arranged in a two-way contingency table. The row variable is TPAHs (*X*) with three categories: Low (L), Medium (M), High (H) and column variable is the sampling points (*Y*) with five categories: Source (S), River (R), 50 m from the Sarno River mouth (50 m), 150 m from the Sarno River mouth (150 m), 500 m from the Sarno River mouth (500 m).

### Log-ratio and log odds ratio analysis

#### Notations

Let **N** = (*n*_*ij*_) be a two-way contingency table that cross-classifies *n* units according to *I* row categories and *J* column categories of *X* and *Y* variables, respectively. Let *X*_*i*_ and *Y*_*j*_ be the i-th and j-th category of *X* and *Y* and let *π*_*ij*_ the probability that *X* = *X*_*i*_ and *Y* = *Y*_*j*_. The matrix of proportions is denoted by **P** = *n*^− 1^**N** with general term *p*_*ij*_. The marginal relative frequencies of the *i-*th row and *j-*th column of **P** are *p*_*i* •_ and *p*_• *j*_ and they may be represented in vector or matrix form. In this paper, the vector **r** (resp. **c**) consists of *p*_*i* •_ (resp. *p*_• *j*_) as elements, while **D**_*r*_ (resp. **D**_*c*_) is the diagonal matrix of these quantities.

Let  be the OR, the complete set of ORs for table **N** is composed by [*I*(*I* − 1)]/2 × [*J*(*J* − 1)]/2 ORs and it can be placed in a two-way table, called , of dimension , where  and .

#### From association model to log Ratio Analysis

The association models (Goodman, 1985) are widely used to analyse two-way contingency tables. The first proposed version was the RC (1) association model (Goodman, 1979), then it was extended to the RC (M) association model to decompose the symmetric association into M components (Goodman, 1985). If *M* = min[(*I* − 1), (*J* − 1)], this model is called saturated. The RC (M) association model is given by


where *μ*_*im*_ and *ν*_*jm*_ are *X*_*i*_ and *Y*_*j*_ scores on dimension *m* (standard coordinates), *φ*_*m*_ is a measure of the strength of the association between *X* and *Y*, *α*_*i*_ and *β*_*j*_ are the main effects of *X* and *Y*, respectively. With respect to the scores, the following constraints are assumed:, and where  and .

Assuming the previous constraints and that the distribution of counts within IJ categories is a multinomial distribution with parameters *n* and *π*_*ij*_, the parameter estimation is computed by the maximum likelihood method.

An alternative estimation method is based on the least square procedure. Let *N*_*ij*_ ~ Po(*nπ*_ij_ = *τ*_*ij*_) be a random variable, if we perform the logarithm transformation we obtain the difference log(*N*_*ij*_/*n*) − log(*π*_*ij*_).

Replacing the random variable with its sample values and considering the RC (M) association model we have


Substituting the probabilities with observed frequency, taking into account the constraints and the condition, we estimate the parameters log(*β*_*j*_) and log(*α*_*i*_) as follows: and .

The estimation of the bilinear part is obtained through the least squares method (D’Ambra, 1988; Escoufier and Junga 1986), minimizing the quantity


where .

We have noted that *a*_*ij*_ is equivalent to the residual of the two-way analysis of variance. The same matrix **A** = (*a*_*ij*_), used in RC (M) association model, is analysed in Log-ratio analysis (Greenacre, 2009). Greenacre, starting from Correspondence Analysis (CA) and using Box-Cox transformation of  (with *α* → 0) applied a SVD on the following matrix


where *L* means logarithm transformation. Based on the different centring system, a comparison among CA, weighted LRA, and RC (M) has been done (Greenacre and Lewi 2009). For analogical criteria the weighted system of weighted LRA is the same of CA. This choice could be justified in a better way as follows.

Considering *N*_*ij*_, when *τ*_*ij*_ → + ∞, then  is a random variable with normal standard distribution^a^. If we consider the random variable , applying the Taylor series, we can say that  provides a useful approximation to  when , thus . Observing that , it follows that , then *E*[log(*N*_*ij*_)] ≅ log(*τ*_*ij*_) and *Var*[log(*N*_*ij*_)] ≅ 1/*τ*_*ij*_. Under the independence hypothesis *τ*_*ij*_ can be estimated by *np*_*i* •_*p*_• *j*_, justifying the weighting system based on row and column marginal totals of **P.**

For the foregoing the association between the categories of *X* and *Y* variables could be studied, by performing a SVD of the double-centred matrix **Z** with respect to *p*_*i* •_ and *p*_• *j*_, : where *M* = *rank*(**Z**) = min[(*I* − 1), (*J* − 1)] with **U**^*T*^**U** = **V**^*T*^**V** = **I** where **u**_*m*_ is the m-th column of **U**, **v**_*m*_ is the m-th column of **V** and the singular values down the diagonal of **Λ** are in descending order *λ*_1_ ≥ *λ*_2_≥.... ≥ *λ*_*M*_. The total variance in weighted LRA can be written in terms of the complete set of the log ORs


The principal and standard coordinates for rows and columns are computed as follows:

, , , .

Setting **r** = (1/*I*)**1** and **c** = (1/*J*)**1** we obtain the unweighted Log Ratio Analysis (Aitchison 1990)^b^:


The LRA decomposes Altham’s measure for *Q* = 2 and the complete set of ORS, in fact:


Althman’s index is an association measure based on the ORs. It can also be defined on the local odds ratio and spanning cell odds ratios. For a deep discussion of this measure, see Edwardes and Baltzan (2000).

#### Weighted LRA properties

The weighted LRA preserves the underlying properties the fundamental characteristics of classical CA: coordinates properties, distance measures, a reconstitution formula, rank of decomposed matrix. It is a powerful tool for analysing compositional data (Aitchison and Greenacre 2002). The weighted LRA to the specific case of log ORs has been introduced. As in RC (M) models (de Rooij and Heiser 2005) the row and column coordinates of the weighted LRA satisfy the following two important properties:
12

Where *d*^2^(**f**_*i*_, **g**_*j*_) is the squared Euclidean distance between the points with coordinates **f**_*i*_ and **g**_*j*_ on the m dimensions. Thanks to these properties the factorial representation of the weighted LRA can be explained both in terms of inner product rule (type I), and distance rule (type II). For type I representation, at least one coordinate set should be drawn using vectors, and the points of the other set projected on these vectors to represent the relationship. For type II the categories for both sets can be represented by points in Euclidean space, with the distance between the points describing the relationship between categories of two sets. These properties permit to visualize in the same factorial plan the categories and the log-ORs. Unfortunately, these important properties do not work for unweighted LRA.

Let  and  be the baseline of row and column score vectors, respectively. Substituting these baselines for the average with respect to *i* and *j*, in this case zero vectors ( and ), and taking into account formula (1), the OR of the pair of categories ij*-th* respect the baseline (Eshima et al., 2001) can be defined


This OR is theoretical and could be interpreted as the contribution of the pair of categories ij-*th* towards the association between X and Y variables. Considering log transformation and using vectors, the previous quantity can be written as


Denoting by  the matrix of dimension *I* × *J*, whose generic element is , we have 

In order to compute a synthesis measure of the complete set of ORs, the OR mean (Me) can be calculated by using formula (2)


Replacing *f*_*i* ' *m*_ and *g*_*j* ' *m*_ by means with respect to *i’* and *j’*, in this case zero vectors, we have:


Using standard coordinates we obtain
3

In the RC (M) model, this quantity is expressed by the Kullback–Leibler information (Eshima et al., 2001). This property is also true for the weighted LRA:


This quantity shows the departure of the assumption of independence. As *Me*(*OR*_*i*0*j*0_) is expressed by the Kullback–Leibler information, the larger this mean is, the stronger the association between *X* and *Y* is. Dividing this mean by the sum of singular values, an index for studying the relationship between the X and Y variables based on the log ORs is obtained:


while the quantity  represents the contribution of the *m*-th pair of coordinate vectors to the relationship between the X and Y variables.

#### From LRA to log-odds Ratio Analysis

The weighted LRA permits the indirect representation of the log ORs. Starting from the de Rooij and Anderson approach (2007) we have proposed a methodology based on the singular value decomposition of log OR matrix for obtaining its direct representation. Summary measures of association were also obtained. Unlike de Rooij and Anderson, the method has been applied to the matrix with the complete set of log odds ratios because, as seen later, it is linked to LRA (see below).

Let *L*(**S**) be a two-way table of dimension  containing the complete set of log ORs, in this table the rows (resp. columns) are formed by all pairs of categories of X (resp. Y). Let **B** and **D** be two square diagonal matrices of dimensions  and  respectively with general terms  and  Performing a SVD of L(**S**) with the matrices **B** and **D,** we have:


We have called this analysis Unweighted Log Odd Ratio analysis (ULORA). ULORA is linked to unweighted LRA, and, consequently, is joined with the Altman measure:


This method does not take into account the weight structures of the rows and columns. Let  and  be two square diagonal matrices of dimensions  and  respectively with general terms *p*_*i* •_*p*_*i* ' •_ and *p*_• *j*_*p*_• *j* '_. Performing a SVD of *L*(**S**) with the matrices  and  we get a weighted analysis of the log OR matrix (WLORA):


The coordinates are:

We obtain a factorial representation in which pairs of categories of X and Y are drawn. Following this approach, at least one coordinate set should be drawn using vectors, and the points of the other set projected on these vectors to represent the weighted ORs. In this case we show that:


Therefore both the weighted LRA and ULORA decompose a synthetic measure of the log ORs. This last one is a weighted version of Altham’s measure.

## Results and discussion

The association between TPAHs (*X*) and sampling points (*Y*) is significative, with Pearson’s chi-squared equal to 687.017. The complete set of log ORs is computed (Table 1).

**Table 1:** **Complete set of log ORs**

	SR	S50m	S150m	S500m	R50m	R150m	R500m	50m150m	50m500m	150m500m
LM	3.296	4.171	4.394	1.399	0.875	1.099	−1.897	0.223	−2.773	−2.996
LH	2.187	2.854	3.045	−1.268	0.667	0.858	−3.455	0.191	−4.123	−4.313
MH	−1.109	−1.317	−1.350	−2.668	−0.208	−0.241	−1.558	−0.033	−1.350	−1.317

The log ORs are very different from 0, therefore the association is confirmed. The synthesis of the complete set of log ORs can be performed computing the Altham index and formula (3), the results are 0.72158 and 0.62545, respectively.

**Figure 1:**
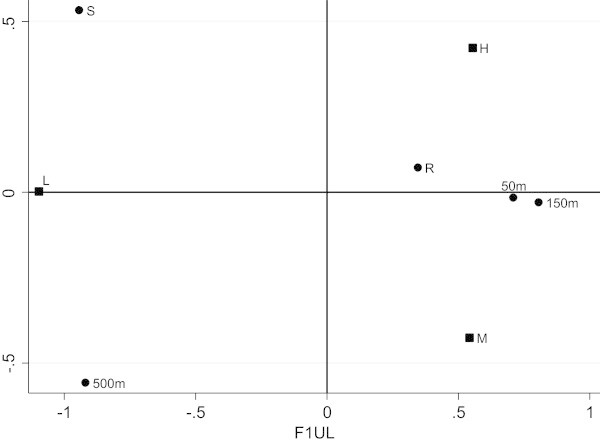
**Factorial representation for unweighted LRA.**

**Figure 2:**
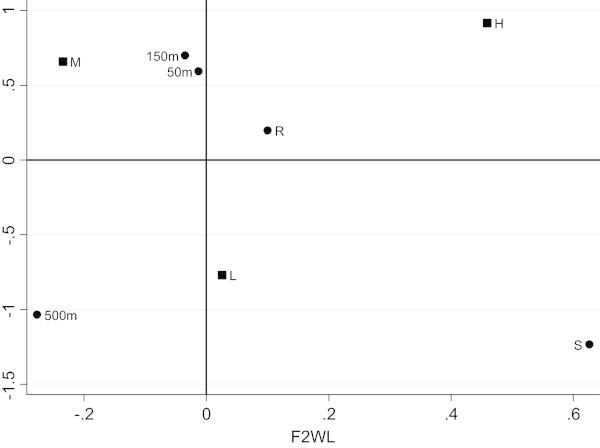
**Factorial representation for weighted LRA.**

Afterwards the unweighted and weighted LRA of the two-way contingency table have been performed. The number of dimensions to be retained is two and the factorial representations have been presented (Figure 1, Figure 2). In these representations we have the categories of X and Y. In Figure 1 we can observe that on the first axis there is a juxtaposition between a low level of pollution and a medium-high level of pollution, with “Source” and “500 meters” associated with a low level of pollution and the other categories of X (“River”, “50 meters” and “150 meters”) linked with a medium-high level of pollution. Figure 2, instead, appears more readable and interesting thanks to the effect of the weighting system. In fact “Source” and “500 meters” remain at a low level of pollution, but the other group is further divided in two more homogeneous groups: “River” associated with a high level of pollution and “50-150 meters” with a medium level of pollution.

**Figure 3:**
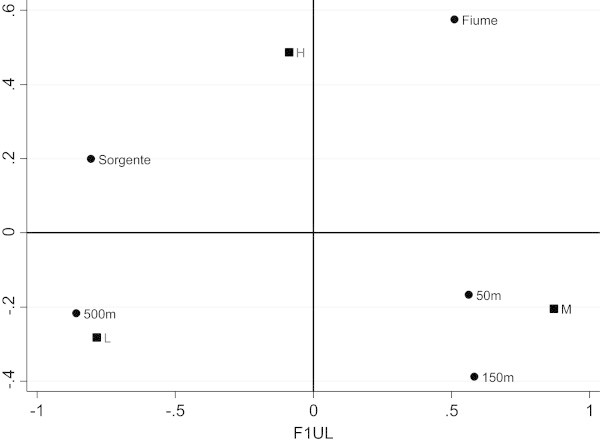
**Factorial representation for unweighted LRA (South-West).**

**Figure 4:**
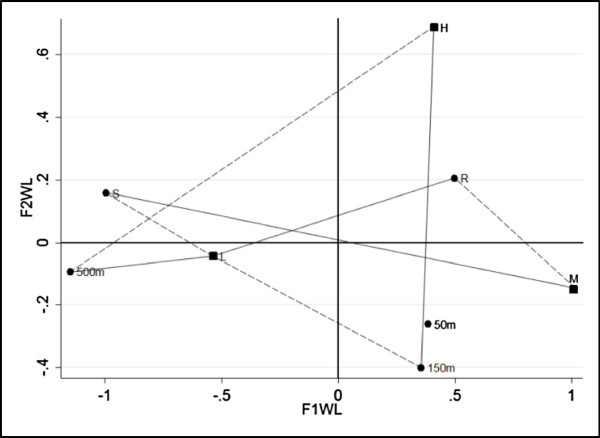
**Factorial representation for weighted LRA (South-West).**

In order to improve the data analysis we have divided the data table into three sub-tables, in which the last three categories of X have been further subdivided into three sub-levels concerning the direction of detection (North-West, West, South-West). This division was made because it was found that the level of pollution of the sea is influenced by the direction of detection. In this paper we have showed only the direction South-West (for the other analysis the authors can be contacted). In factorial representation of unweighted LRA (Figure 3) three associations are clear: “500 meters” with a low level of pollution, “50 and 150 meters” with a medium level of pollution and “River” with a high level of pollution. The position of category “Source” is ambiguous, in fact it is in the middle between low and high level of pollution. In our opinion, this ambiguity depends on the different values of the margins of the data table. In order to take into account this feature of the data, the weighted LRA, that allows us to include a system of weights, has been performed. The factorial representation (Figure 4) is better, in fact the classification of the ambiguous category “Source” has been resolved and is correctly associated with a low level of pollution. As the marginal relative frequencies of data table (rows: 0.614, 0.284, 0.102; columns: 0.149, 0.419, 0.145, 0.143, 0,144) are different, then weighted LRA is preferred. Moreover, for weighted LRA, the indirect representation of log-ORs can be appreciated. For example considering log *OR*_*L*,*M*;*S*,*R*_ and log *OR*_*L*,*H*;*R*,500_, according to formula (2) the log-OR depends on the length of the distances between categories. In our case log *OR*_*L*,*M*;*S*,*R*_ is clearly greater than 1, since the solid lines are much longer than the dotted ones, for the second log *OR*_*L*,*H*;*R*,500_ the contrary happens therefore it is smaller than 1. We fitted the RC (2) association model using the marginal proportions as weights. The results are very similar to those obtained by the weighted LRA.

Subsequently to study the association between X and Y we have considered the matrix of the complete set of log ORs (Table 2).

**Figure 5:**
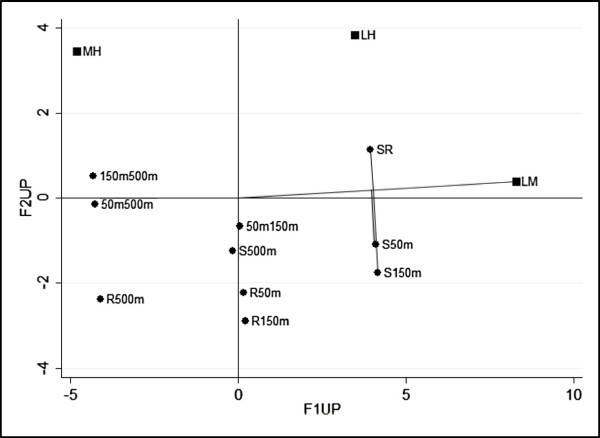
**Factorial representation for ULORA (South-West).**

**Figure 6:**
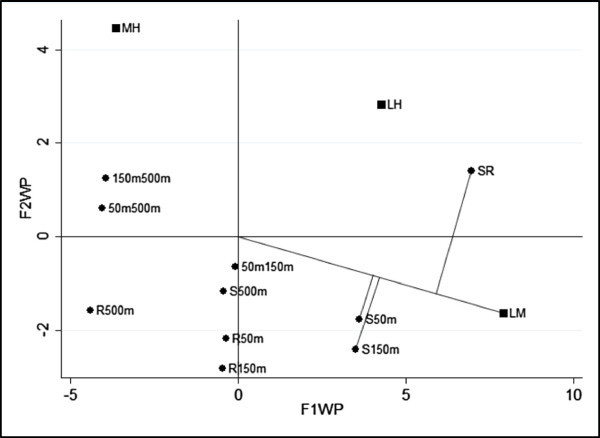
**Factorial representation for WLORA (South-West).**

The ULORA and WLORA have been performed. The factorial representations, on retained axes, are in Figure 5 and Figure 6. At first glance, the factorial representations look very similar but differences exist, as a matter of fact in Figure 6 the category “SR” and “MH” have coordinates greater than in Figure 5.

**Table 2:** **Complete set of log ORs (South-West)**

	SR	S50m	S150m	S500m	R50m	R150m	R500m	50m150m	50m500m	150m500m
LM	3.296	3.255	3.255	−0.223	−0.041	−0.041	−3.520	0.000	−3.477	−3.477
LH	2.187	0.588	0.118	−0.981	−1.599	−2.069	−3.168	−0.470	−1.569	−1.099
MH	−1.109	−2.668	−3.137	−0.758	−1.558	−2.028	0.351	−0.470	1.910	2.380

This is a consequence of the system of weights; in fact in WLORA we have taken into consideration the marginal relative frequencies of the rows and the columns as a weighting system. When there are large differences among the marginal relative frequencies of the rows or of the columns, it could be relevant to take this information into account, therefore the WLORA is preferred. ULORA and WLORA permits the visualization of the log-ORs through the projection of the column points onto the row vectors (or viceversa). For example, we have focalized our attention on three log-ORs computed using the same row vector: log *OR*_*L*,*M*;*S*,*R*_, log *OR*_*L*,*M*;*S*,50_ and log *OR*_*L*,*M*;*S*,150_. The projections are different in the two analysis depending on the system of weights. In Figure 6 we can see that there is a strong association between the categories “Source-River” and Low-High level of pollution, “Source-50 meter” and Low-Medium level of pollution and “Source-150 meter” and Low-Medium level of pollution. This association can be justified both by the proximity of the categories and through the log-ORs (represented by projections of the first category on the second). Moreover, in this case it is also possible to make an interpretation in terms of variations. In fact, the log *OR*_*L*,*H*;*S*,*R*_, tells us that when you switch from “Source” to “River” is very likely that the level of pollution jumps from Low to High, the same happens when we switch from category “Source” to “50 meters”, where it is very likely that the pollution goes from low to medium. Therefore, given the sequence of the measured points, it is possible to argue that passing from “River” to see the pollution decreases from high to medium.

## Conclusions

In this paper we discussed the RC (M) association model and weighted LRA providing a justification for the logarithm transformation and weighting system. RC (M) association models and LRA are based on Newton–Raphson (NR) algorithm and the SVD, respectively. The convergence of the NR depends on the starting point. On the contrary the SVD is extremely stable and computationally simpler. Regarding the number of dimensions, the LRA allowed to choose the number of dimensions to be retained later. A criterion could be the variance explained by the first components. Another criterion could be the application of a bootstrap or jackknife procedure for verifying the stability of singular values. In the RC (M) association models, for a given dimensionality_,_ main effects and interaction terms were estimated. Then we extended LRA to the study of log-ORs, obtaining the indirect representation of the log ORs and its synthesis measures. Finally we applied the SVD to the unweighted and weighted Log-ORs matrix (ULORA and WLORA) obtaining a direct representation of log-ORs. We also got summary measures of association. The ULORA and WLORA were applied to the complete set of log-ORs for linking these methods to LRA.

In the further study we intend to extend the introduced methodologies to three-way contingency table (Gallo and Simonacci 2013), to the other types of ORs (i.e. cumulation, continuation and global) and to the ratio of two contingency tables. Considering that the two contingency tables are of same dimensions: one representing the target population, *X*_*ij*_ the second a subset of this population with a specific character, *Y*_*ij*_. Let *X*_*ij*_ ~ Po(*τ*_*ij*_) and *Y*_*ij*_|*X*_*ij*_ = *k*_*ij*_ ~ Bin(k_ij_, *p*_*ij*_) be. One demonstrates *Y*_*ij*_ ~ Po(*τ*_*ij*_*p*_ij_). Then the methodologies presented could be extended to the analysis of ratios *r*_*ij*_ = *y*_*ij*_/*x*_*ij*_.

## Endnotes

^a^A continuity and symmetry correction can also be applied when one discrete distribution is approximated by the normal distribution.

^b^Other weight systems can be applied, for example *p*_*i* •_ + *p*_• *j*_ for squared tables.
